# Epidemiology, Distribution, Key Characteristics, and Challenges of Candidozyma auris (Formerly Candida auris): A Narrative Review With a Special Focus on Türkiye

**DOI:** 10.7759/cureus.107778

**Published:** 2026-04-27

**Authors:** Rasim Gökmen, Ayşegül Erdoğan, Birsen Cunetoğlu

**Affiliations:** 1 Public Health, Dulkadiroğlu District Health Directorate, Kahramanmaraş, TUR; 2 Public Health, Kahramanmaraş Sütçü İmam University, Kahramanmaraş, TUR; 3 Infectious Diseases, Adana City Training Hospital, Adana, TUR

**Keywords:** candida auris, candidozyma auris, fungal drug resistance, invasive candidiasis, public health surveillance

## Abstract

This narrative review summarizes the epidemiology, microbiological and clinical features, antifungal resistance, transmission dynamics, and public health significance of *Candidozyma auris* globally and with a focus on Türkiye. *C. auris* has emerged as an important fungal pathogen because of its capacity for healthcare-associated colonization, environmental persistence, biofilm formation, laboratory misidentification, and multidrug resistance. Available evidence suggests that its rapid global spread is related to environmental tolerance, skin colonization, interclade phenotypic differences, and antifungal resistance mechanisms. Reported cases from Türkiye further support the need for strengthened infection control and surveillance systems. It represents a significant nosocomial fungal threat that necessitates the simultaneous implementation of clinical management and public health responses. In addition, it has been observed that the dominant clade in Türkiye is Clade I, that early cases were misidentified due to laboratory method-related limitations, and that there are substantial variations in antifungal susceptibility even within the same case series.

## Introduction and background

*Candidozyma auris (*formerly* Candida auris*) is an emerging yeast pathogen first identified in 2009 from the external auditory canal of a patient in Japan [1]. Subsequent retrospective analyses identified earlier isolates dating back to 1996 that were later confirmed as *C. auris* [2]. Following its initial identification in Asia, it was progressively reported from other continents [3]. By 2016, cases had been documented in multiple regions, including India, South Africa, Kuwait, and the United States [4-7].

Similar to its rapid global spread, once introduced into a healthcare setting, *C. auris* can quickly disseminate across surfaces, medical equipment, and the hands of healthcare personnel, persist for prolonged periods, and cause outbreaks [8]. Unlike other *Candida* species, *C. auris* frequently exhibits resistance to multiple antifungal classes, significantly complicating treatment. Reported mortality rates for invasive *C. auris* infections have ranged from 30% to 60% across published studies, although these estimates vary according to patient populations, disease severity, and study design. Collectively, these features establish *C. auris* as a major global public health threat that has attracted global attention from health authorities [9,10].

International public health authorities have classified *C. auris* as a priority threat. The Centers for Disease Control and Prevention (CDC) designated *C. auris* as an “urgent global health threat” and included it among the most dangerous antimicrobial-resistant pathogens in the 2019 Antibiotic Resistance Threats in the United States report [11]. Similarly, the World Health Organization (WHO), in its first list of priority fungal pathogens published in October 2022, categorized *C. auris* in the critical priority group. According to WHO, invasive fungal infections represent a growing public health concern due to increasing resistance and limited treatment options, with *C. auris* recognized as one of the most critical pathogens in this context [12].

The genus *Candida* has historically encompassed a diverse group of yeast-form species that do not constitute a single phylogenetic lineage; rather, it is a polyphyletic genus, meaning that its members do not share a recent common ancestor. With advances in genomic studies, many species previously classified under *Candida* have been reassigned to different genera. In a phylogenomic study published by Liu et al. in 2024, *Candida auris* and related species were shown to form a distinct lineage within the Metschnikowiaceae family, separate from the classical *Candida* species of the Debaryomycetaceae family. Based on these genomic findings, the genus *Candidozyma* was proposed for this clade [13]. Although the name *Candida* remains widely used due to historical and clinical convention, the taxonomically accurate designation is now *Candidozyma auris*. Accordingly, recent guidelines and publications refer to the organism as *Candidozyma auris* [14-16]. In this report, *C. auris* refers to both names and the same organism.

To provide a clearer clinical and public health perspective, this review first summarizes the global emergence and epidemiology of *C. auris*, and then discusses its microbiological and clinical features, antifungal resistance profile, diagnostic challenges, and infection control implications, with particular emphasis on the available evidence from Türkiye. In this context, the objectives of this review are: (i) to summarize the global epidemiology and key microbiological and clinical features of *C. auris*, (ii) to describe the epidemiological characteristics and current clade distribution of cases reported from Türkiye, (iii) to evaluate diagnostic challenges and antifungal resistance patterns in light of global data and findings from Türkiye, and (iv) to discuss the implications of the available evidence for surveillance, infection control, and clinical management in Türkiye.

Search strategy and literature selection

This study was designed as a narrative review. A focused literature search was conducted to identify relevant publications on *C. auris*, particularly regarding epidemiology, microbiological and clinical features, antifungal resistance, diagnosis, infection control, and reports from Türkiye. Searches were performed in PubMed, Scopus, Web of Science, and Google Scholar, as well as in up-to-date guidelines, surveillance reports, and official documents published by the WHO, CDC, European Centre for Disease Prevention and Control (ECDC), and the Turkish Ministry of Health. The search process was conducted iteratively from February 3, 2026, to April 3, 2026, with the final literature update performed on April 3, 2026. The search strategy used the terms “Candida auris” or “Candidozyma auris” in combination with keywords related to Türkiye or Turkey, epidemiology, antifungal resistance, diagnosis, infection control, and surveillance. Retrieved sources were screened at the title, abstract, and full-text levels, and English-Turkish language original studies, reviews, case series, guidelines, and official reports that were directly relevant to the aims of the review, particularly those providing up-to-date information and data related to Türkiye, were included. Reference lists of key articles were also screened to identify additional relevant sources. Because of the narrative design and the heterogeneity of the available literature, no formal systematic screening protocol, risk of bias assessment, meta-analysis, or pooled quantitative analysis was performed.

## Review

Epidemiology

Global Emergence and Clade Structure

The emergence and global spread of *C. auris* have been investigated through genomic analyses, which suggest that this pathogen emerged independently and nearly simultaneously across multiple continents around the 2010s. These studies have identified distinct clades separated by 1000s of single-nucleotide polymorphisms (SNPs), each associated with specific geographic regions. A strong phylogeographic structure has been demonstrated, leading to the classification of clades as the South Asian Clade (Clade I), East Asian Clade (Clade II), South African Clade (Clade III), and South American Clade (Clade IV) [17].

Subsequently, additional clades have been identified. In 2018, isolates from Iran were found to represent a distinct lineage, separated from the previously described clades by more than 200,000 SNPs and designated as Clade V (Iranian Clade). More recently, in 2024, the Indomalayan Clade (Clade VI) was described, differing from its closest clade by more than 37,000 SNPs [18]. In addition to their genetic divergence, clades also exhibit differences in pathogenicity and virulence characteristics [19].

Taken together, these findings support the hypothesis that *C. auris* did not originate from a single geographic source but rather emerged through parallel evolutionary processes in multiple regions, followed by global dissemination [17,20].

Global Spread and Regional Trends

According to published global reports, *C. auris* infections have been identified in more than 40 countries worldwide since its first description in 2009 [21]. A marked increase in reported cases has been observed, particularly after 2015. According to CDC data, *C. auris* was first detected in the United States in 2016 and subsequently spread rapidly, with a total of 3,270 cases reported by 2021. The number of clinical cases increased from 476 in 2019 to 1,471 in 2021, representing more than a threefold rise. The most pronounced increase occurred in 2020-2021, and it has been suggested that challenges associated with the COVID-19 pandemic, such as hospital overcrowding and disruptions in infection control practices, may have facilitated the spread of *C. auris* [22,23].

A similar upward trend has been observed in Europe. While only sporadic cases were reported between 2013 and 2017, ECDC issued a rapid risk assessment for *C. auris* in 2018, highlighting the increasing number of cases. Published European reports indicate that the total number of reported *C. auris* cases reached 655 during 2020-2021 [8,24]. Hospital outbreaks have been reported in countries such as the United Kingdom, Spain, Italy, and Germany, while sporadic cases have been documented in several other European countries [25-29]. In Türkiye, the first cases of *C. auris* fungemia were reported in 2020 from hospitals in Istanbul [30-32].

Pandemic-Associated Acceleration

The COVID-19 pandemic has had a notable impact on the epidemiology of *C. auris* since 2019. Data from the United States indicate that the surge in *C. auris* cases during 2020-2021 was partly associated with the pandemic. In 2020, *C. auris* was reported for the first time in eight additional states, followed by initial reports in three more states in 2021. However, since 2022, the annual rate of increase in *C. auris* cases in the United States has relatively slowed [22,33].

Geographic Distribution and Regional Heterogeneity

The global burden of *C. auris* is unevenly distributed. While only sporadic imported cases have been reported in some countries, others have experienced sustained endemic transmission or large-scale outbreaks. For example, only a limited number of cases have been documented to date in Canada and Scandinavian countries, whereas in countries such as South Africa, India, the United States, and Spain, case numbers have reached levels that can be considered endemic. These differences may partly reflect variations in surveillance capacity and diagnostic awareness across healthcare systems, as well as intrinsic characteristics of *C. auris*.

In addition, the geographic distribution has been influenced by the predominance of specific clades in different regions. For instance, the East Asian Clade is generally less resistant and has shown more limited spread compared to other clades, whereas the South Asian Clade and the South American Clade have disseminated across broader geographic areas [34].

Surveillance

With the rapid global spread of *C. auris*, international surveillance has become increasingly important. Many countries have added *C. auris* to their list of notifiable diseases or issued specific alerts. Organizations such as the WHO and ECDC have called on countries to share case notifications and implement standardized diagnostic and screening protocols [12,14]. In Europe, ECDC has established a platform for cross-country information sharing; when a new *C. auris* clade emerges, or a panresistant strain is identified in a country, other countries are informed through this system [14]. WHO has also advocated for the establishment of a global surveillance network for fungal infections [12]. In the United States, the CDC requires that cases of *C. auris* be reported promptly to local and state health authorities as well as to the CDC. Additionally, the CDC provides nationwide surveillance data through its online platform “Tracking *C. auris*”, reporting confirmed cases by year and by state [33,35].

Because *C. auris* can rapidly colonize patients in healthcare settings and often remains asymptomatic, screening of contacts and high-risk individuals is essential. Screening is typically performed using superficial swab cultures. The CDC recommends collecting swabs from the axilla and groin simultaneously, followed by inoculation onto selective media or analysis using polymerase chain reaction (PCR) methods [36].

Microbiology

Taxonomy and Morphology

C. auris is a yeast-form fungus belonging to the phylum Ascomycota. It is phylogenetically closely related to the *Candida haemulonii* complex. Based on recent phylogenomic data, the species has been reclassified within the genus *Candidozyma* in the Metschnikowiaceae family [8,13].

*C. auris* is a haploid fungus and, similar to some other *Candida* species, may exhibit dimorphism. Under laboratory conditions, it has been shown to grow both as round to oval budding yeast cells and as aggregate forms resembling pseudohyphal or filamentous structures [37]. Unlike classical *Candida* species, it rarely forms true hyphae or pseudohyphae, which can make microscopic identification more challenging. In contrast to *Candida albicans*, it does not produce germ tubes. Cells are typically observed singly or in pairs and do not form chains. Colony morphology may vary between strains; some appear dry and matte, whereas others are more moist and sticky. These differences reflect intraspecies variability [38,39].

Growth and Reproduction

*C. auris* is a yeast that reproduces by budding and proliferates asexually. It can multiply rapidly under favorable conditions, with growth influenced by factors such as temperature, nutrient availability, and humidity. In healthcare settings, it can quickly colonize human skin and the surfaces of medical devices. This rapid colonization contributes to prolonged carriage and increases the risk of transmission. Isolates are also capable of forming biofilms within a short period. This indicates not only quantitative growth but also structured organization, which contributes to persistence and complicates eradication [38,39].

Environmental Tolerance

One of the most notable features of *C. auris* is its environmental resilience. It can survive at temperatures up to 42°C and in salt concentrations of up to 10%. While most *Candida* species cannot tolerate such conditions, the persistence of *C. auris* under these environmental stresses complicates infection control efforts [38-40]. This characteristic has been proposed as a potential adaptation to climate change. Notably, *C. auris* did not emerge from a single geographic region and subsequently spread worldwide; rather, it appeared simultaneously across multiple regions. One hypothesis suggests that global warming may have facilitated the breach of the “endothermy barrier,” which typically protects humans from environmental fungal pathogens. According to this view, *C. auris* may represent one of the first fungal pathogens to emerge in humans as a consequence of climate change [38].

*C. auris* can persist for prolonged periods in warm and humid environments, on medical device surfaces, bedside areas, monitors, air conditioning filters, and even cleaning equipment. Many commonly used surface disinfectants may be ineffective against *C. auris* [39,41,42].

Virulence Factors

*C. auris* possesses several virulence factors that contribute to its pathogenicity. One of the most important is a species-specific adhesin, the surface colonization factor (Scf1), which facilitates adherence to surfaces. Through these adhesins, *C. auris* can form biofilms, colonize surfaces, and enhance virulence in invasive infections. Biofilms are structured communities of microorganisms that provide protection against external stressors. This organization not only promotes resistance to antifungal agents but also facilitates transmission. Certain strains are known to secrete enzymes such as proteases, lipases, and phospholipases, which can damage host tissues. In addition, efflux pumps present in resistant strains enhance the extrusion of antifungal agents from the cell. Collectively, these factors increase both the ability of *C. auris* to cause infection and its resistance to treatment [38,39].

Many of the virulence factors of *C. auris* should not be considered as independent features but rather as components of an integrated set of biological traits associated with persistent skin colonization, immune evasion, and environmental resilience. Understanding how mechanisms such as adhesion, metabolic adaptation, biofilm formation, and antifungal resistance interact within the context of host-pathogen interactions is essential for explaining the rapid epidemiological spread and resistance patterns of this emerging pathogen [43].

Clinical features

Risk Factors and Clinical Findings

*C. auris* infection is primarily healthcare-associated and is most commonly observed in critically ill patients, particularly those in intensive care units. The most frequently reported risk factors include intensive care unit admission, prolonged hospitalization, the presence of central venous catheters and other invasive devices, mechanical ventilation, exposure to broad-spectrum antibiotics or antifungals, parenteral nutrition, severe comorbid conditions, and immunosuppression. In a recent systematic review and meta-analysis, the pooled prevalence of diabetes mellitus among patients with confirmed *C. auris* infection was reported as 36%, and the presence of diabetes was associated with *C. auris* infection with an odds ratio of 1.40. In the same study, all-cause mortality among patients with both *C. auris* infection and diabetes was reported as 57%. These findings suggest that diabetes is not merely a comorbidity but also a relevant marker for risk stratification and screening prioritization. The same meta-analysis identified chronic kidney disease, hypertension, and respiratory diseases as common comorbidities, while key healthcare-associated risk factors included central venous catheterization, intensive care unit stay, and mechanical ventilation [43,44]. Similar risk factors, particularly central venous catheterization and mechanical ventilation, have also been reported in pediatric cases [45].

*C. auris* infections can present with a wide and variable clinical spectrum. Patients may be colonized without exhibiting any clinical signs or symptoms. Infections may remain localized or rapidly progress to invasive disease, including sepsis. Therefore, early diagnosis and prompt intervention are critical in individuals infected with *C. auris* [38,39].

Invasive *C. auris* infections most commonly present as candidemia. These infections typically develop during hospitalization and are often associated with a severe clinical course with an increased risk of organ failure. However, other clinical manifestations may also occur, including wound infections, urinary tract infections, meningitis, peritonitis, and, rarely, endocarditis. There are no specific clinical signs unique to *C. auris* infection. Patients generally present with features similar to bacterial sepsis or invasive infections caused by other *Candida* species, including fever, hypotension, tachycardia, and altered mental status [38,39].

Most patients are asymptomatically colonized prior to the development of infection. Unlike *Candida* species such as *C. albicans* and *C. glabrata*, which predominantly colonize the gastrointestinal tract, *C. auris* most commonly colonizes the skin, particularly the nares, axilla, and inguinal regions, without causing clinical symptoms. This asymptomatic colonization represents a significant risk for transmission even in the absence of active infection. Colonized individuals can facilitate the spread of the organism within healthcare settings through contaminated surfaces and medical equipment, contributing to transmission to other patients and healthcare personnel. This characteristic plays a key role in the outbreak potential of *C. auris* [38,39,41].

Another important aspect of *C. auris* infections is their high mortality rates. Mortality varies depending on factors such as the patient's overall health status and the presence of comorbid conditions [38,39]. Several studies have reported in-hospital mortality rates ranging from 30% to 70% in cases of *C. auris* candidemia [37]. Although this wide range reflects differences in patient populations and disease severity, overall mortality can be considered substantial. For example, in a multicenter cohort study conducted in the United States, 54.6% of 187 patients with *C. auris* candidemia died [46].

In addition, the ability of *C. auris* to persist on the skin for prolonged periods, combined with the difficulty of eradication and ease of transmission, underscores the necessity of strict infection control measures [40,47].

Diagnostic Methods and Challenges

*C. auris* has a high rate of misidentification due to its microbiological similarity to certain uncommon *Candida* species. It can grow on routine culture media, such as Sabouraud agar, but cannot be reliably distinguished from other *Candida* species based on morphology alone. In laboratories relying on conventional diagnostic methods, *C. auris* is frequently misidentified as species such as *C. haemulonii*, *Candida lusitaniae*, or *Candida parapsilosis* [39].

Automated microbiological identification systems, such as VITEK 2 (bioMérieux SA, Marcy-l'Étoile, France), API ID (bioMérieux SA), and Phoenix (Becton, Dickinson and Company, Franklin Lakes, New Jersey, United States), have limited databases and may not reliably distinguish *C. auris* from closely related species. Therefore, especially in cases involving resistant strains, the use of advanced diagnostic techniques is required for accurate species identification [38,39].

Accurate identification of *C. auris* can be achieved using matrix-assisted laser desorption ionization time-of-flight mass spectrometry (MALDI-TOF MS). The two most commonly used MALDI-TOF systems, Bruker Biotyper (Bruker Corporation, Billerica, Massachusetts, United States) and VITEK MS (bioMérieux SA), have incorporated *C. auris* into their databases. In addition, laboratories can reliably identify *C. auris* by sequencing the D1-D2 region of the 28S ribosomal DNA or the internal transcribed spacer (ITS) regions of ribosomal DNA [38,39].

However, access to diagnostic platforms capable of accurate identification remains limited in many centers in Türkiye. These diagnostic gaps pose significant risks not only at the individual patient level but also for public health. If a patient is misidentified and appropriate isolation measures are not implemented, the patient may remain in clinical wards without adequate infection control precautions, facilitating transmission to other patients. This represents a serious concern with the potential to lead to healthcare-associated outbreaks [42].

Antifungal Resistance Profile

One of the most notable characteristics of *C. auris* is its widespread and multidrug antifungal resistance. Global analyses indicate that approximately 50% of *C. auris* isolates are resistant to at least one antifungal class, and although less common, panresistant strains resistant to all three major antifungal classes have been reported [8]. Echinocandin antifungals, including anidulafungin, micafungin, and caspofungin, are considered the most effective agents against *C. auris*. However, in recent years, resistance to this class has also been reported. This resistance is typically associated with mutations in the *FKS* gene region, which alter the structure of the target enzyme and reduce drug binding, potentially leading to treatment failure. Echinocandins are recommended as first-line therapy for C. auris infections. In cases of inadequate clinical response, second-line agents such as liposomal amphotericin B may be considered. For infections caused by resistant strains, combination therapies (e.g., echinocandin plus amphotericin B) have been used in some centers; however, evidence supporting their efficacy remains limited [38-40]. Heterogeneous susceptibility may reflect methodological variability or emerging resistance; clinicians should interpret susceptibility results in conjunction with clinical response.

New therapeutic agents are also under investigation. Rezafungin, a long-acting echinocandin, and ibrexafungerp, an orally available triterpenoid glucan synthesis inhibitor, are currently being evaluated for their clinical efficacy and safety. In infections caused by echinocandin-resistant or multidrug-resistant strains, fosmanogepix, an agent that disrupts cell wall mannoprotein synthesis via inhibition of Gwt1 and is currently in phase three clinical trials, has emerged as a promising treatment option [38,39]. Decolonization is not recommended [38,42].

Interclade Differences

Because *C. auris* has emerged globally as genetically distinct clades, efforts have focused on identifying clade-specific molecular and phenotypic characteristics. One of the main differences lies in antifungal susceptibility profiles. For example, isolates from the Clade II are generally more susceptible to fluconazole, with some strains remaining responsive. In published clade-based analyses, Clade I isolates have typically shown fluconazole resistance exceeding 90%, whereas reported amphotericin B resistance has ranged from 30% to 50%. For Clades III and IV, echinocandin resistance has been estimated at approximately 5%-7% [8]. Consistently higher minimum inhibitory concentration (MIC) values observed in Clade III and IV isolates have raised clinical concerns regarding treatment efficacy. In addition to antifungal susceptibility, experimental models suggest that virulence may also vary between clades. For instance, studies have shown that Clade IV isolates may be associated with higher mortality in animal models compared to Clade II isolates, which appear to have relatively lower pathogenicity [19].

*Candidozyma auris* in Türkiye

Initial Reports and National Surveillance

The first reports of *C. auris* cases in Türkiye were published in 2020 from hospitals in Istanbul [30-32]. Subsequently, the first cases in Anatolia were identified in 2022 [48,49]. Over time, the number of reported cases has increased, and C. auris has become an important healthcare-associated pathogen in Türkiye [42].

According to ECDC data, a total of 114 cases of *C. auris* infection or colonization were reported in Türkiye between 2020 and 2023 [15]. In addition, the Ministry of Health collects data on *C. auris* within the framework of the National Healthcare-Associated Infections (HAI) Surveillance system and evaluates these data through the Infection Prevention and Control Advisory Board. In addition, to better assess infections and colonization outside the scope of HAI surveillance, a “Non-HAI *C. auris* Notification Form” was introduced in September 2024. This form is intended to enable the monitoring of screening results as well [42]. In addition, three-month surveillance data (September 1 to November 30, 2024) collected through the National HAI Surveillance system and the Non-HAI *C. auris* Notification Form were presented in the “*Candida auris* in Healthcare Settings” guideline prepared by the General Directorate of Public Health. During this three-month period, a total of 515 C. auris cases were reported, of which 237 were classified as HAI and 278 as non-HAI cases. These notifications originated from 87 hospitals for HAI cases and 59 hospitals for non-HAI cases, with the majority of reporting hospitals (n=69) located in Istanbul. When unit types were analyzed, 81.6% of cases were reported from intensive care units [42].

Early Diagnostic Challenges

In the initial reported cases, identification methods such as API 20C AUX, API ID 32C, and VITEK 2 yielded incorrect results, leading to delays in appropriate treatment. Accurate identification was later achieved using MALDI-TOF. These findings suggest that *C. auris* was likely misidentified and underrecognized in Türkiye during earlier periods, and that its presence may date back further than initially reported [50].

Antifungal Resistance Patterns and Clade Profile

Analysis of antifungal resistance profiles in these initial cases demonstrated resistance to fluconazole and amphotericin B, with preserved susceptibility to echinocandins [30,31,51]. This pattern is consistent with Clade I. In addition, whole-genome sequencing (WGS) based phylogenetic analysis of cases reported from Istanbul in 2021 confirmed that the isolates belonged to Clade I [52].

In published Turkish series, fluconazole resistance has been reported at very high rates, in some reports approaching universal resistance, whereas amphotericin B resistance has also been frequently observed. In contrast, susceptibility to echinocandins is generally preserved; however, heterogeneous responses and emerging resistance, particularly to caspofungin, have been described in some studies. Additionally, the presence of panresistant strains and the development of resistance during therapy represent significant challenges in clinical management [52-55].

From a diagnostic perspective, antifungal susceptibility results may vary depending on the method used. In particular, the reliability of the VITEK 2 system for amphotericin B and fluconazole has been reported to be limited, requiring confirmation with reference methods [56]. At the molecular level, isolates have been found to be consistent with Clade I. However, the presence of resistant phenotypes even in isolates without detectable mutations in classical resistance genes suggests that additional, yet uncharacterized mechanisms may contribute to antifungal resistance [53,57].

Clinical and Epidemiological Features of Reported Cases

Epidemiological data indicate that *C. auris* is particularly prominent among intensive care unit patients. In one study of candiduria cases, concurrent candidemia was reported in 72% of patients in whom *C. auris* was detected in urine cultures, suggesting that urinary isolation may, in some cases, indicate concurrent invasive infection [58]. In candidemia cohorts, *C. auris* has been associated with prolonged intensive care unit stays, invasive procedures, use of broad-spectrum antibiotics, and the presence of indwelling catheters [59,60].

In terms of clinical outcomes, some studies have reported that mortality associated with *C. auris* is comparable to or lower than that observed with other *Candida* species, emphasizing that mortality is primarily related to the severity of the underlying illness. However, achieving microbiological eradication has emerged as one of the most important factors associated with a significant reduction in mortality [59,60].

When studies reported from Türkiye are evaluated collectively, *C. auris* demonstrates a heterogeneous and complex profile in terms of both clinical course and control. Regarding environmental resistance, the susceptibility of clinical isolates to biocides varies between strains; notably, reduced efficacy has been observed with quaternary ammonium compounds, whereas sodium hypochlorite at concentrations ≥0.5% has been shown to be the most reliable fungicidal agent [61].

Public health measures and infection control

There is no standardized decolonization protocol that has been seen to reliably eliminate *C. auris *colonization. Although in vitro activity against antiseptics such as chlorhexidine has been reported, their clinical effectiveness in reducing skin colonization burden and preventing transmission has not been systematically demonstrated; therefore, routine decolonization is not recommended. However, in the context of high-risk procedures, selected patient populations, or ongoing transmission despite other infection prevention and control measures, chlorhexidine-based approaches may be considered on a case-by-case or institutional basis. Accordingly, the core strategy includes early diagnosis, implementation of contact precautions, environmental disinfection, and appropriate equipment decontamination [37,42,62].

Rooms of patients with *C. auris* should be cleaned at least once daily and undergo thorough “terminal cleaning” after discharge. Terminal cleaning should include not only floors but also beds, furniture, bathrooms, curtains, and other surfaces. Some commonly used hospital disinfectants are ineffective against *C. auris*. In particular, products containing only quaternary ammonium compounds have been shown to be insufficient for eradication. For example, benzalkonium chloride-based disinfectants, although effective against many bacteria and *Candida* species, may not completely eliminate *C. auris*. Therefore, CDC and ECDC recommend the use of Environmental Protection Agency (EPA) approved disinfectants listed under List P for *C. auris*. If such products are not available, disinfectants with proven sporicidal activity, including agents comparable to those listed under EPA List K, may be considered. In the absence of these options, locally licensed products with equivalent active ingredients may be used. These typically include sodium hypochlorite (bleach) or peroxide-based agents. Chlorine solutions at 1000 ppm (0.1%) or higher and disinfectants containing 1% peracetic acid have been shown to be effective even against biofilms. Combination approaches may further enhance efficacy. Some studies have demonstrated that sequential use of sodium hypochlorite followed by hydrogen peroxide vapor can achieve complete eradication of *C. auris* from various surfaces. “No touch” disinfection technologies, such as ultraviolet-C (UV-C) irradiation systems and vaporized hydrogen peroxide (VHP), have also been evaluated. While UV-C may be less effective against *Clostridioides difficile* spores, it can still reduce environmental burden. Shared equipment represents an important transmission vector. Devices such as glucometers, blood pressure cuffs, stethoscopes, pulse oximeter probes, and mobile workstations can harbor *C. auris*. Therefore, all equipment must be disinfected with appropriate agents after each use. Whenever possible, equipment should be dedicated to a single patient; otherwise, high-level disinfection between patients is required. Clear institutional responsibilities for cleaning and disinfection must be defined, as ambiguity in these roles has been identified in outbreak investigations. Accordingly, all cleaning protocols should be standardized in written form, reinforced through staff training, and, when feasible, monitored using objective tools such as fluorescent markers or ATP-based assessment [39,41,42,62-65].

Table 1 summarizes the findings regarding C. auris globally and from Türkiye, as found in this review.

**Table 1:** Summary of key findings on Candidozyma auris globally and in Türkiye

Categories	Global findings	Findings from Türkiye
Emergence and spread	First identified in 2009, *C. auris *appears, based on genomic data, to have emerged independently and nearly simultaneously across different continents, followed by rapid global spread	The first cases in Türkiye were reported from Istanbul in 2020, and a marked increase in reported cases has been observed in subsequent years
Clade structure	Six major clades have been described, differing in geographic distribution, virulence, and antifungal susceptibility	Available molecular data indicate that reported isolates in Türkiye are predominantly associated with Clade I
Environmental persistence	*C. auris* can persist for prolonged periods on skin, environmental surfaces and medical devices; biofilm formation contributes to persistence and difficulty of eradication	Studies from Türkiye have reported reduced susceptibility to some biocides in certain strains, while sodium hypochlorite has been shown to be a more reliable fungicidal agent
Identification	*C. auris* is frequently misidentified by conventional laboratory systems; MALDI-TOF MS and sequencing provide more accurate identification	In some of the first reported Turkish cases, isolates were initially misidentified by API- and VITEK-based systems, and correct identification was later achieved by MALDI-TOF
Antifungal resistance	Fluconazole resistance is very high globally. Amphotericin B resistance has also been reported in many regions, while echinocandin resistance, although less common, has increasingly drawn attention	In reported Turkish series, fluconazole resistance is very high, amphotericin B resistance is frequent and echinocandin susceptibility is generally preserved but heterogeneous
Susceptibility testing	Antifungal susceptibility results may vary according to the testing method used, leading to differences in interpretation for some agents	Data from Türkiye suggest that, for some antifungals, VITEK 2 results may not fully agree with reference methods
Risk profile	Intensive care unit stay, invasive procedures, central venous catheters, mechanical ventilation, antimicrobial exposure and severe comorbidities are major risk factors	Reported cases in Türkiye have occurred predominantly in intensive care patients; invasive procedures, catheter use and broad-spectrum antimicrobial exposure have been prominent
Colonization and transmission	Asymptomatic skin colonization facilitates nosocomial spread through healthcare workers’ hands, medical equipment and environmental surfaces, increasing outbreak potential	Reported cases from Türkiye indicate a marked risk of colonization and in hospital spread, particularly in intensive care settings
Clinical pattern	Candidemia is the most common invasive presentation; wound infections, urinary tract infections, peritonitis, meningitis and other invasive or non-invasive manifestations may also occur	In some Turkish series, candiduria has been reported together with candidemia, suggesting that urinary isolation may, in some cases, indicate concurrent invasive infection
Clinical outcome	Mortality is high, but outcomes are largely influenced by the severity of underlying illness and the ability to control the infection	Studies from Türkiye suggest that microbiological eradication may be associated with better clinical outcomes
Surveillance	Notification, screening and interinstitutional information sharing are key components of control strategies	In Türkiye, in addition to national HAI surveillance, a non-HAI *C. auris* notification form was introduced in 2024
Infection control	Contact precautions, appropriate patient placement, daily and terminal cleaning and effective surface disinfection are central control measures	The national guideline recommends structured environmental cleaning and the use of chlorine, hydrogen peroxide and peracetic acid based agents

Limitations

This is a narrative review and was not conducted using a systematic methodology. Literature selection may therefore be subject to selection bias. In addition, language bias may have resulted in the underrepresentation of Turkish language studies not indexed in major international databases. The included studies are heterogeneous in terms of design, populations, outcomes, and laboratory methods, which limits direct comparability. Because of this substantial heterogeneity, no pooled quantitative analysis of mortality, resistance, or other outcomes was undertaken. Data from Türkiye are largely derived from case reports, case series, and single-center retrospective studies, and the absence of patient-level data precluded any pooled risk analysis. Therefore, estimates of mortality, antifungal resistance frequencies, and other clinical outcomes should be interpreted with caution and should not be considered precise national estimates. Furthermore, variability in diagnostic methods and antifungal susceptibility testing complicates the interpretation of findings. Due to unreported or misidentified cases, the true disease burden may also be underestimated.

Recommendations

Access to MALDI-TOF and molecular diagnostic methods should be expanded to improve diagnostic accuracy. Standardized methods should be used for antifungal susceptibility testing, and critical results should be confirmed. Active surveillance and screening should be implemented in intensive care units. Infection control measures, including isolation, contact precautions, and environmental cleaning, should be standardized and consistently implemented. National surveillance systems should be strengthened. Rational use of antifungal agents should be promoted.

Future directions

Multicenter and prospective studies are needed in Türkiye. Molecular epidemiology and WGS-based studies should be expanded. Resistance mechanisms should be investigated in greater detail. The relationship between colonization and invasive infection should be clarified. The effectiveness of infection control interventions should be evaluated.

## Conclusions

*C. auris* has emerged as an important nosocomial pathogen globally and in Türkiye because of its increasing incidence, multidrug resistance, environmental persistence and close association with healthcare related transmission. Available data from Türkiye indicate that reported cases occur predominantly in intensive care units, are largely associated with Clade I, and remain difficult to manage because of high fluconazole resistance, frequent amphotericin B resistance and diagnostic challenges. These findings suggest that the epidemiological and clinical significance of *C. auris* in Türkiye extends beyond individual case management and should be considered within a broader infection prevention and public health framework.

Effective control therefore requires early diagnosis, reliable laboratory identification, appropriate antifungal therapy, active surveillance and standardized infection control measures. Because early cases in Türkiye were initially misidentified by conventional laboratory systems, expanding access to MALDI-TOF MS and molecular diagnostic methods is essential. In addition, given the predominance of Clade I and the very high fluconazole resistance reported in Turkish isolates, empirical fluconazole therapy should be avoided in suspected *C. auris* infections unless susceptibility is confirmed. The concentration of cases in high risk hospital units further underscores the need to strengthen national surveillance, laboratory capacity and coordinated control strategies. Moreover, as the available data are largely based on case reports and single center series, there is a clear need for multicenter studies in Türkiye to better define the epidemiology, resistance patterns and clinical outcomes of *C. auris* infections.
